# Predictive Factors and Risk Mapping for Rift Valley Fever Epidemics in Kenya

**DOI:** 10.1371/journal.pone.0144570

**Published:** 2016-01-25

**Authors:** Peninah M. Munyua, R. Mbabu Murithi, Peter Ithondeka, Allen Hightower, Samuel M. Thumbi, Samuel A. Anyangu, Jusper Kiplimo, Bernard Bett, Anton Vrieling, Robert F. Breiman, M. Kariuki Njenga

**Affiliations:** 1 Global Disease Detection Division, United States Centers for Disease Control and Prevention-Kenya, Nairobi, Kenya; 2 Ministry of Agriculture, Livestock and Fisheries, Nairobi, Kenya; 3 Division of Parasitic Diseases and Malaria, Centers for Disease Control and Prevention, Atlanta, Georgia, United States of America; 4 Paul G. Allen School for Global Animal Health, Washington State University, Pullman, Washington, United States of America; 5 Ministry of Health, Nairobi, Kenya; 6 International Livestock Research Institute, Nairobi, Kenya; 7 Faculty of Geo-Information Science and Earth Observation (ITC), University of Twente, Enschede, The Netherlands; Division of Clinical Research, UNITED STATES

## Abstract

**Background:**

To-date, Rift Valley fever (RVF) outbreaks have occurred in 38 of the 69 administrative districts in Kenya. Using surveillance records collected between 1951 and 2007, we determined the risk of exposure and outcome of an RVF outbreak, examined the ecological and climatic factors associated with the outbreaks, and used these data to develop an RVF risk map for Kenya.

**Methods:**

Exposure to RVF was evaluated as the proportion of the total outbreak years that each district was involved in prior epizootics, whereas risk of outcome was assessed as severity of observed disease in humans and animals for each district. A probability-impact weighted score (1 to 9) of the combined exposure and outcome risks was used to classify a district as high (score ≥ 5) or medium (score ≥2 - <5) risk, a classification that was subsequently subjected to expert group analysis for final risk level determination at the division levels (total = 391 divisions). Divisions that never reported RVF disease (score < 2) were classified as low risk. Using data from the 2006/07 RVF outbreak, the predictive risk factors for an RVF outbreak were identified. The predictive probabilities from the model were further used to develop an RVF risk map for Kenya.

**Results:**

The final output was a RVF risk map that classified 101 of 391 divisions (26%) located in 21 districts as high risk, and 100 of 391 divisions (26%) located in 35 districts as medium risk and 190 divisions (48%) as low risk, including all 97 divisions in Nyanza and Western provinces. The risk of RVF was positively associated with Normalized Difference Vegetation Index (NDVI), low altitude below 1000m and high precipitation in areas with solonertz, luvisols and vertisols soil types (p <0.05).

**Conclusion:**

RVF risk map serves as an important tool for developing and deploying prevention and control measures against the disease.

## Introduction

The Rift Valley fever (RVF) epidemics occur every 3 to 10 years in specific regions of the Greater Horn of Africa, southern and western Africa, and in the Arabian Peninsula, resulting in high morbidity and mortality among livestock and humans [1–5]. A prediction model for RVF epidemics that is based on global climatic patterns is used to forecast outbreaks with some success, although the sensitivity and specificity of the model can be improved by inclusion of additional indicators such livestock herd immunity levels, vector profile, and geologic and geographic factors associated with high risk regions [6–8]. Experts agree that the severity of RVF epidemics can be reduced by recognition of early warnings followed by rapid implementation of prevention and control measures [9,10]. In 2008, international experts and decision-makers from eastern Africa developed a risk-based decision support tool designed to guide responses during various stages of the RVF disease cycle [9]. The tool was based on 12 stages of an RVF cycle that start with the inter-epidemic period and continue to the post-epidemic recovery [9].

Of the countries affected by RVF, Kenya has reported the largest number of epidemics involving both humans and livestock [11]. Retrospective analysis of the livestock surveillance data in the country identified a pattern of the disease spread across the country following the first detection in the 1930s, and culminating in the current situation where up to 55% of the administrative districts in the country were involved in the 2006/2007 RVF epidemic (13). Here, we used semi-quantitative and statistical risk assessment methodologies to develop a RVF risk map that determines risk of RVF epizootics for each of the 391 administrative divisions in the country (based on the 1999 administrative map). Exposure risk was evaluated by determining the number of times a division had been involved in epizootics, whereas the outcome risk was assessed using the prevalence data collected during the last outbreak (2006–2007) in both humans and animals. In addition to risk classification, we determined the climatologic, geologic, and geographical factors associated with risk of RVF outbreaks and used the predicted probabilities to develop a RVF risk map for Kenya.

## Materials and Methods

### RVF Surveillance Data

Surveillance records on RVF outbreaks covering the period 1951 to 2007 were obtained from the Kenya Department of Veterinary Services, Ministry of Agriculture, Livestock and Fisheries, Kenya Medical Research Institute, and United States’ Centers for Disease Control and Prevention, Kenya. These records identified the year and the number of months each outbreak occurred as well as administrative units (province, district, and division) affected. An RVF outbreak was defined as above normal occurrence of abortions, perinatal mortality and hemorrhagic syndrome in livestock with or without human involvement. Outbreaks events involving 1 or 2 districts were considered as localized outbreaks. A national RVF outbreak was declared when more than 2 administrative districts were reporting RVF disease in livestock. Most primary cases in livestock were screened by a combination of post mortem and serological testing; however in recent years reverse transcription polymerase chain reaction testing was also used for confirmation before official declarations of disease. The data were cleaned for statistical analysis and collapsed by administrative division and in some cases districts, which were regarded as reliable spatial units of analysis. The analysis was based on the administrative map used during the 1999 human population and housing census, comprising of 69 districts and 391 divisions of Kenya (as existing in 1999). To develop the risk maps, both a semi-quantitative and statistical risk modeling of RVF outbreaks were carried out.

### Semi-Quantitative Risk Modeling

To characterize the likelihood of RVF occurrence in each of 69 districts and 391 divisions, the risk of exposure and outcome were assessed in order to classify the district and division as having a higher or lower likelihood of RVF disease occurrence in relation to the other districts and divisions in the country. Ultimately, the risk profile for each district and division was based on assessment of risk of exposure and severity of outcome, and additional input from human and animal health experts (expert group discussion).

#### Risk of exposure

The risk of exposure among susceptible human and animal populations in a district was assessed using retrospective surveillance data of the disease in the area as previously reported [11]. Briefly, the risk of RVF exposure in a district was determined as the proportion (in percent) of the national RVF epizootic years that the district was involved in an outbreak since the first report of the virus in the district. Using these surveillance data, the proportional measures of exposure were ranked and the 50^th^ percentile determined to be 40% probability of exposure. All districts with a proportion of ≥ 40% were assigned a score of 3, those with a proportion of >1% - 39% assigned a score of 2, and districts that have never reported RVF disease assigned a score of 1.

#### Risk of outcome

Data on animal and human disease from the 2006/07 RVF outbreak in Kenya were used to assess the outcome of disease in both animals and humans. During the 2006/07 outbreak, extensive surveillance in animals was carried out in 54 of 69 districts and results of RVF sero-surveys (IgM and IgG assays) used as a measure of the outcome of RVF disease in each district [12]. Anti-RVF virus IgM and IgG sero-prevalence and RT-PCR results from 27 districts that provided samples were included [12]. The sero-prevalence was ranked and the 50^th^ percentile identified. Districts that reported a prevalence ≥ 3.3% and ≥ 7.2% for IgM and IgG respectively were assigned a score of 3, >0–3.2 and >0–7.1 were assigned a score of 2, and districts that have reported 0% or no samples were tested for RVF were assigned a score of 1. The sero-prevalence for IgM and IgG were considered separately and later averaged to yield one estimate of the severity of the disease in animals for each district.

The number of laboratory confirmed human cases from the 2006/07 outbreak for each districts was obtained [10] and used as a measure of the outcome of the disease in humans in each district. The laboratory confirmed human cases were reported from 13 districts and since disease in humans is typically preceded by disease in animals, districts that reported human cases were given a score of 3 while those that did not report any human cases were assigned a score of 1.

Finally, the scores for severity of disease in animals and humans were averaged to yield an outcome estimate for each district. To obtain a total weighted estimate for each district, the exposure and outcome risk scores were multiplied, resulting in a (maximum score of 9). The resulting scores were then used to categorize a district as having high (score ≥5), medium (score = ≥2 to <5), or low (< 2) likelihood of RVF outbreak.

#### Expert Group Discussions

The outputs from the exposure and outcome risk assessments were subjected to scrutiny in a 3-day workshop attended by 47 livestock and medical officers drawn from all the 8 provinces of Kenya. The workshop systematically discussed the occurrence of RVF disease in every division seeking to identify: (i) districts and divisions where RVF occurrence had been reported over the years, (ii) districts and divisions that had RVF outbreaks but the data was missing owing to ineffective or non-presence of livestock disease surveillance, and (iii) divisions within the high and medium risk districts where RVF was not likely to occur due to factors such as absence of livestock (for example national parks and game reserves, mountains), (iv) outcome risk using severity of the disease during the last two epidemics (the 1997/98 and 2006/07) that may not have been captured in the data used for assessing outcome risk. The outcome of the focus group discussions were added to the risk classification of districts and divisions to achieved the final risk classification. Finally, a spatial map of the risk profile at division level was created.

### Statistical Risk Modeling

We use statistical modeling to identify geologic, geographic, and demographic predictor variables important for RVF.

#### Data Sources

Using data from the 2006/07 RVF outbreak in Kenya, predictor variables for the risk of RVF outbreaks were determined through statistical modeling. The list of putative variables tested for their associations with the RVF outbreak are presented in Table 1. Data on livelihood zones were obtained from the Famine Early Warning Systems Network (FEWS NET, http://www.fews.net/Pages/default.aspx). FEWS NET has classified geographical areas into homogeneous units where people share similar livelihoods including options for obtaining food, income and market opportunities to inform food security analyses. For the purpose of these analyses, levels of livelihood zones were collapsed into five categories based on their association with the outcome. Livelihood categories that had significant association with RVF were identified and kept as distinct categories while those that had insignificant association with RVF were collapsed into a single category and used as the reference category.

**Table 1:** List of putative risk factors tested for their relationship with Rift Valley Fever outbreaks.

Variable	Description
Livelihood zones	Livelihood practices (2006), FEWS NET (http://www.fews.net/Pages/default.aspx)
Land cover	Global land cover data (GLC 2000), FAO–collapsed into 6 land use types (cultivated, herbaceous cover, tree cover, mosaic, and water)
NDVI	Monthly average, minimum, maximum values from: 1999–2010, SPOT VEGETATION
Human population	Human and household census for 1960, 1970, 1980, 1990, 1999, 2009; Kenya National Bureau of Statistics
Elevation	Consortium for Spatial Information, Shuttle Radar Topography Mission (CSI SRTM) data at 1 km resolution using the soil type and texture layer
Soil types	FAO’s Harmonized World Soil Database (HWSD), 2008, FAO
Livestock data	Gridded Livestock of the World 2.0 and Livestock Geowiki data sets
Precipitation	Tropical Rainfall Measuring Mission (TRMM), 0.5°: Monthly average for the period 1997–2013
	Climate Prediction Centre Merged Analysis of Precipitation (CMAP), 2.5° Monthly average for the period 1979–2011

The global land cover data (GLC2000) developed by the European Commission was used [13]. These data are gridded at 1 km^2^ spatial resolution. Overall, the country has 11 types of land cover. For the purpose of this analysis, the land cover types were collapsed including into 6 inlcuding artificial, cultivated, herbaceous cover, tree cover, mosaic and water. The most dominant land cover type at the administrative division level assumed to represent the entire division. Livestock data were obtained from FAO’s Gridded Livestock of the World database [14]. It comprised of standardised global and sub-national distribution maps (at a 5 kilometer resolution) for the main livestock species including cattle, buffalo, sheep, goats, pigs, chickens and other poultry [14]. For this analysis the species of livestock considered included cattle, sheep, goats and camels. Data on human population collected in 1960, 1970, 1980, 1990, 1999 and 2009 were obtained from the Kenya National Bureau of Statistics. Two precipitation products were used: one from Tropical Rainfall Measuring Mission (TRMM) and the other from Climate Prediction Centre Merged Analysis of Precipitation (CMAP) (Table 1). Normalized Difference Vegetation Index (NDVI) data for the period 1999 to 2010 were obtained from SPOT VEGETATION (http://free.vgt.vito.be/). NDVI, a measure of amount and vigor of green vegetation on land surface, is derived from red and near infrared reflectance measurements. Its values range between -1 and 1.0; negative values indicate the presence of clouds and water, positive values near zero indicate bare soil and higher values indicate dense vegetation. To reduce cloud effects in the NDVI imagery, daily SPOT VEGETATION images are aggregated into 10-day composites where for each pixel the value with the maximum NDVI value is selected. To diminish remaining atmospheric effects, we applied an iterative Savitzky-Golay filter to the temporal series of each pixel [15]. We spatially aggregated the data per division and subsequently extracted minimum, average and maximum values for each division.

NDVI extracts are available on 10 day-intervals at a spatial resolution of 1 km. For this study, minimum, maximum and average values for each division were extracted.

The elevation data used in this analysis were obtained from the Consortium for Spatial Information of the Consultative Group of the International Agricultural Research (CGIAR-CSI; website http://www.cgiar-csi.org) at a resolution of 90 m. The data were derived from the Shuttle Radar Topography Mission (SRTM) whereby missing data were interpolated. Data on soil types were extracted from the Harmonized World Soil Database (HWSD) [16]. The data has a resolution of 1 km and over 15000 different soil mapping units are recognized in the database. Attributes used for this study are soil texture and type.

#### Risk Factors Analysis

Descriptive analyses were conducted to determine the distribution of outbreaks by division. Crude associations between outbreaks and categorical predictor variables were determined using Chi square tests. For continuous variables, mean values and their 95% confidence intervals were generated. These models were used to assess linearity assumption for the continuous variables by fitting quadratic functions and determining their significance in the model. Variables that could not satisfy this assumption (e.g. elevation and livestock density) were converted to categorical variables. The goodness of fit of the various forms of the precipitation variables was evaluated based on the likelihood ratio tests. These included monthly values, 1–3 month lagged values, 2–3 month running cumulative and mean values. For NDVI, minimum, mean and maximum values were also evaluated. Forms of precipitation and NDVI variables that gave the largest log likelihood estimates were used in the subsequent analyses.

Multivariable models were fitted to the data using both backward and forward variable selection procedures. One model used Climate Prediction Centre Merged Analysis of Precipitation (CMAP) precipitation while the other used Tropical Rainfall Measuring Mission (TRMM) precipitation products. The goodness of fit of the models was determined using Hosmer-Lemeshow test (with 10 groups). First order interaction terms between variables were also tested. The level of confidence for all these analyses was 95%. Residual analysis was also conducted and scatter plots of standard residuals against fitted values were done to identify any outliers or scenarios that the model could not predict well. A map of predicted probabilities by division was created using Arc GIS version 9.2.

## Results

### Semi-Quantitative Risk Assessment

The probability-impact weighted scoring identified 15 districts as high, 22 medium, and 32 as low risk. However, further analysis of these data by the animal and human health experts resulted in moving of 6 districts from medium to high risk, and 4 districts from low to medium risk, giving a final total of 21 (30.4%) high, 20 (29%) medium, and 28 (40.6%) low risk districts (Table 2). The 28 low risk districts included all 20 districts in former Western and Nyanza provinces, 7 districts in former Rift Valley province, and one district (Nyandarua) in the former Central province.

**Table 2:** List of RVF high and medium risk high districts as assessed by probability-impact scores and the final risk level after input from the animal and experts.

Province	District	Weighted risk estimate score	PI Risk categories	Final risk level
Central	Kiambu	3.8	Medium	High
Central	Maragua	6.8	High	High
Central	Nyeri	4.5	High	High
Central	Thika	6.0	High	High
Coast	Kilifi	6.8	High	High
Coast	Kwale	5.3	High	High
Coast	Malindi	5.0	High	High
Coast	Mombasa	5.3	High	High
Coast	Tana River	4.0	Medium	High
Coast	Taita Taveta	5	High	High
Eastern	Machakos	5.3	High	High
Eastern	MeruCentral	3.5	Medium	High
Nairobi	Nairobi	6.0	High	High
North Eastern	Garissa	6.0	High	High
North Eastern	Wajir	5.5	High	High
Rift Valley	Baringo	6.0	High	High
Rift Valley	Kajiado	5.0	High	High
Rift Valley	Laikipia	3.8	Medium	High
Rift Valley	Nakuru	5.3	High	High
Rift Valley	Trans Nzoia	3.0	Medium	High
Rift Valley	Uasin Gishu	3.0	Medium	High
Central	Kirinyaga	4.0	Medium	Medium
Central	Murang'a	4.0	Medium	Medium
Coast	Lamu	1.0	Low	Medium
Eastern	Embu	3.5	Medium	Medium
Eastern	Isiolo	4.0	Medium	Medium
Eastern	Kitui	4.0	Medium	Medium
Eastern	Makueni	2.0	Medium	Medium
Eastern	Marsabit	2.0	Medium	Medium
Eastern	Mbeere	3.0	Medium	Medium
Eastern	Meru North	1.0	Low	Medium
Eastern	Meru South	3.0	Medium	Medium
Eastern	Moyale	1.0	Low	Medium
Eastern	Mwingi	4.0	Medium	Medium
Eastern	Tharaka	3.0	Medium	Medium
North Eastern	Mandera	3.0	Medium	Medium
Rift Valley	Koibatek	1.0	Low	Medium
Rift Valley	Marakwet	2.0	Medium	Medium
Rift Valley	Narok	2.0	Medium	Medium
Rift Valley	Samburu	3.5	Medium	Medium
Rift Valley	West Pokot	2.0	Medium	Medium

**Fig 1:**
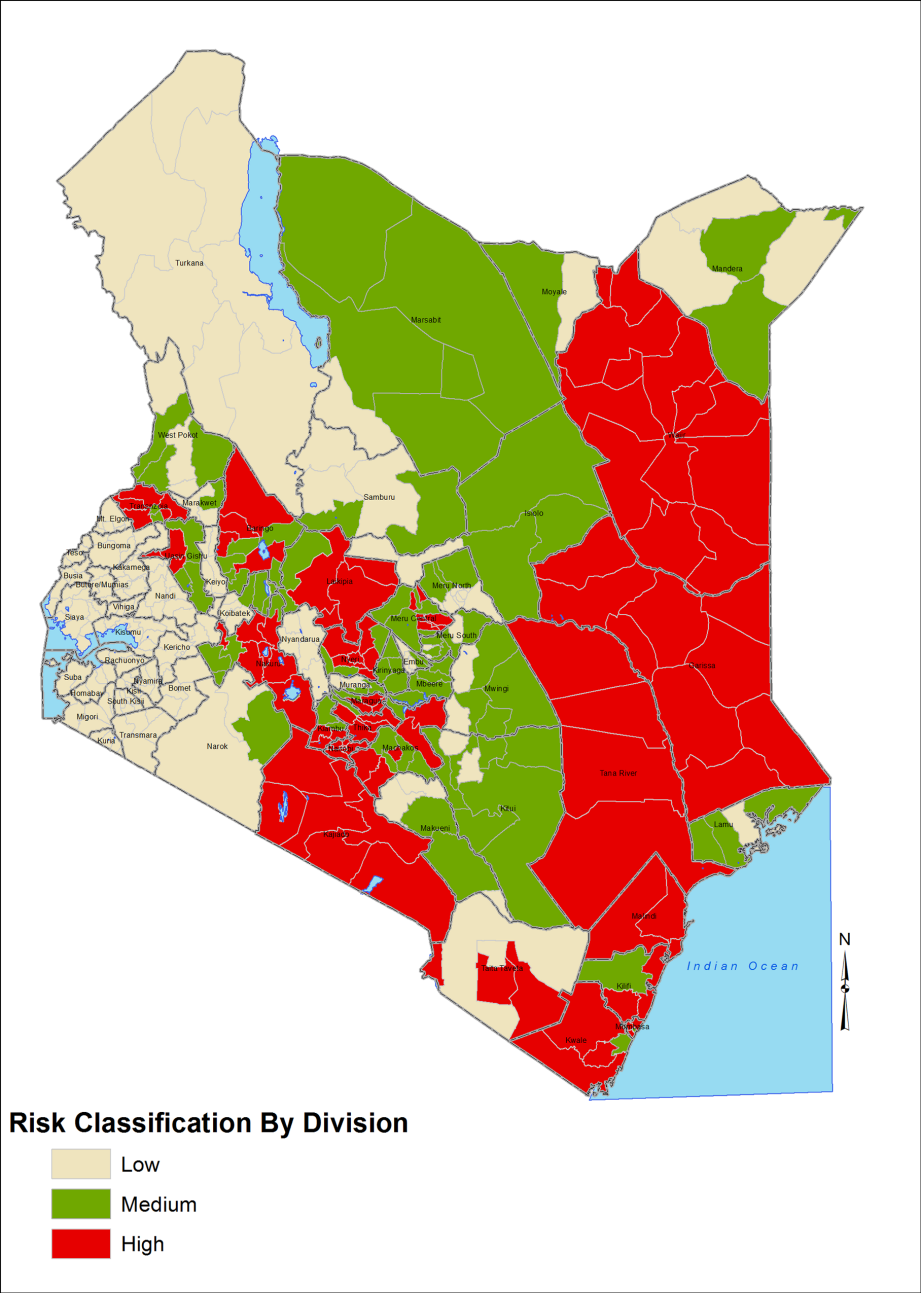
Rift Valley Fever risk map for Kenya, 2012 based on the semi-quantitative risk assessment for likelihood of RVF epizootic and expert opinion.

The experts reviewed the risk level of each administrative division within districts classified as medium and high risk in order to provide a more granular risk map of the disease in the country. The final outcome was a RVF risk map shown in Fig 1. Of a total of 391 divisions in the country, 101 (25.8%) divisions located in the 21 high-risk districts were classified as high risk whereas 100 (25.6%) divisions located in 35 districts were classified as medium risk (Table 2). A total of 190 (48.6%) divisions located in 47 administrative districts were categorized as low risk, including all divisions in Nyanza and Western provinces (Table 2). Divisions within medium or high risk districts that were classified as being at low risk of RVF disease in livestock included game parks and national reserves where livestock are not present. With the exception of Wajir and Garissa districts where all the areas were classified as being at high risk due to the nomadic nature of the livestock keepers who move across the districts, all other high and medium risk districts had certain administrative divisions singled out as having variable likelihood of disease occurrence Fig 1.

Most of the eastern regions of Kenya, including 4 of 7 districts (Kilifi, Kwale, Tana River, and Mombasa) in Coast and 2 of 3 districts (Garissa and Wajir) in Northeastern province, were at high risk for RVF disease occurrence Fig 1. In addition, the districts located in the southern regions of the expansive Rift Valley province, being areas where RVF was originally and most frequently reported have remained at high risk. In contrast, most districts in the Western region of the country, including all districts in Nyanza (N = 12) and Western (N = 8) provinces, and Turkana and Samburu districts in the northern region of Rift Valley province were at low risk of RVF disease Fig 1. The north central and south-central regions of Kenya, including most of the districts in 11 of the 13 divisions in Eastern provinces were at medium risk.

### Predictive Factors of High Risk Divisions

**Fig 2:**
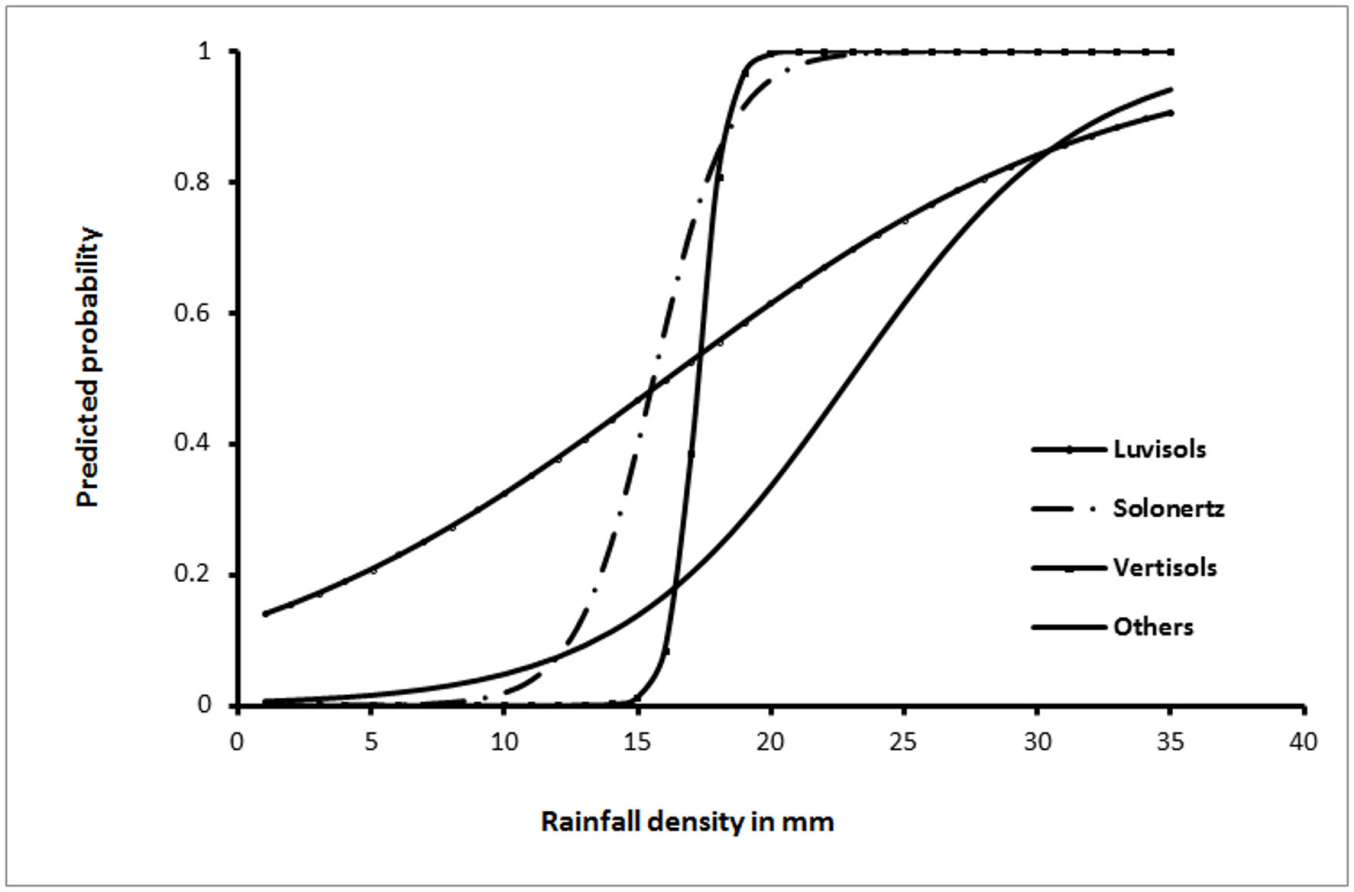
Graphical interpretation of the effects of rainfall and soil type on the risk of RVF outbreak.

The results of the multivariable analyses of the risk factors for RVF outbreaks are presented in Table 3. The final model used precipitation data from the CMAP, which had better fit compared to data from the TRMM. The odds of RVF outbreak increased with increased precipitation, high NDVI values, low altitude and low temperatures. The NDVI did not meet the linearity assumption and it was hence fitted as a quadratic term. The model suggested that there was a significant interaction between soil type and precipitation. This interaction, as outlined in Fig 2, indicates that as the level of precipitation increases, the risk of an RVF outbreak in areas with solonertz and vertisols increases at much faster rate than in areas with other soil types. In addition, the ultimate levels of risk attained in areas with other soil types did not get to those expected in the high-risk areas (with solonertz and vertisols soil types).

**Table 3:** Multivariable logistic regression models fitted to the 2006/2007 RVF outbreak data in Kenya.

Variable	Level	Odds Ratio		P>|Z|
		Estimate	95% CI	
Soil	Luvisols	1.27	0.77–1.85	0.42
	Solonertz	1.82	1.28–2.59	0.01
	Vertisols	1.22	0.70–2.12	0.48
	Others	1.00	-	-
Precipitation (TRMM)		1.09	1.07–1.11	0.00
Luvisols x precipitation		1.11	1.03–1.19	0.00
Solonertz x precipitation		1.16	1.09–1.23	0.00
Vertisols x precipitation		1.11	1.03–1.20	0.01
Elevation	≤1000	1.00		
	>1000 - ≤1500	0.46	0.28–0.75	0.00
	>1500	0.19	0.11–0.33	0.00
NDVI		0.34	0.08–1.39	0.13
NDVI square		11.41	2.21–58.85	0.00
Temperature		0.82	0.79–0.86	0.00

**Fig 3:**
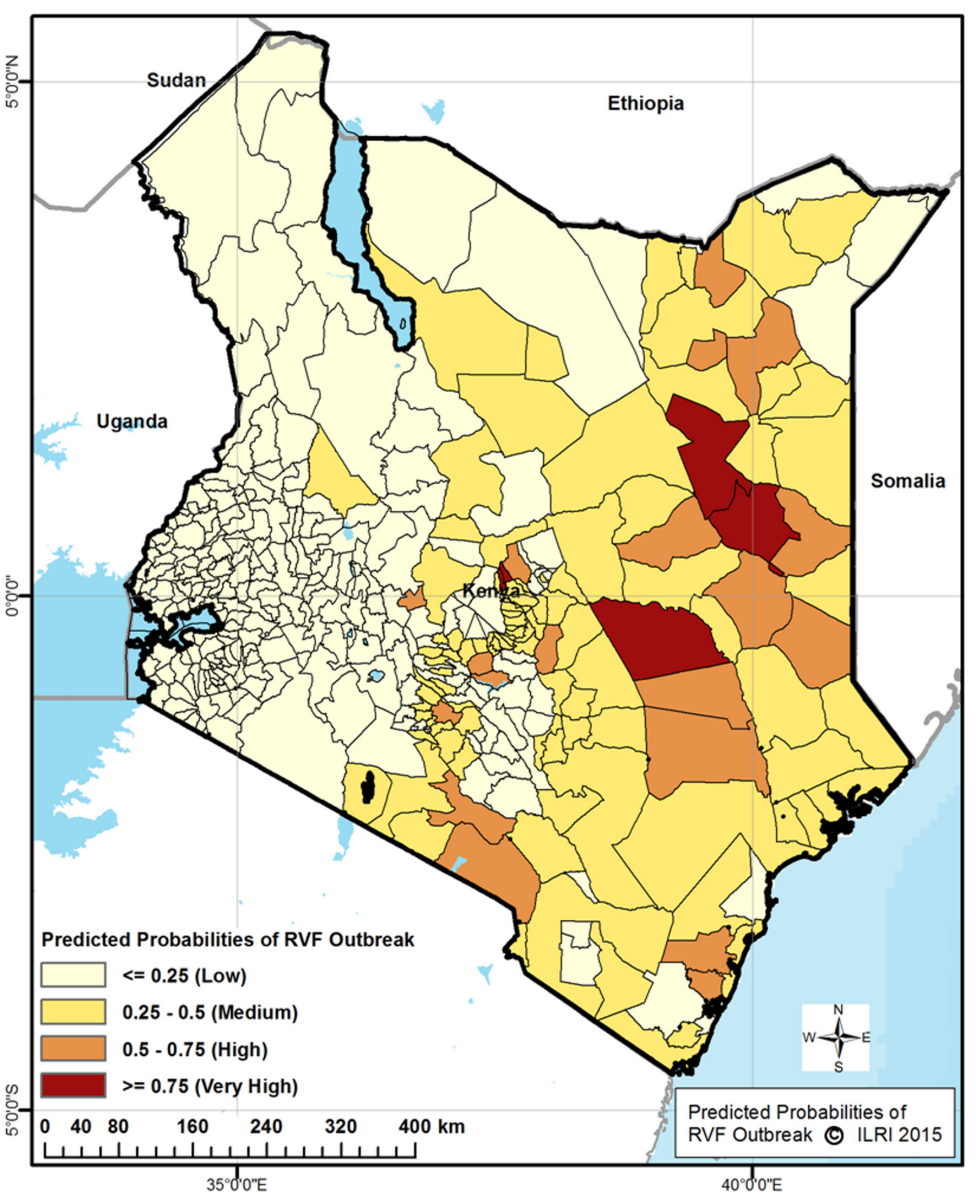
RVF risk map for Kenya generated from predicted probabilities by administrative divisions based on Centre Merged Analysis of Precipitation (CMAP).

Using the predicted probabilities of the factors that remained in the final model as significantly associated with the RVF outbreaks, we generated the RVF risk map for Kenya shown in Fig 3.

Overall, of the 391 divisions, 20 divisions (6.1%) had very high or high predicted probability of RVF occurrence, 100 (25.6) had medium predicted probability, 257 (65.3%) had low predicted probability and 14(3.6%) had very low predicted probability of RVF occurrence. The risk map showed the eastern part of the country to be at medium to high risk of RVF occurrence. Divisions to the western part of the Great Rift Valley were predicted to be at low or very low probability of occurrence of RVF. In addition, divisions in the central highlands and central northern parts of Kenya were predicted to be at low risk of RVF occurrence.

## Discussion

This study applied semi-quantitative risk assessment and statistical modeling techniques to develop RVF risk maps for Kenya, a country that has had the highest number of major RVF epidemics between 1951–2007 [11]. The 2006/07 epidemic was the most extensive affecting 26 of the 69 districts across six of the eight provinces in Kenya, with the highest livestock morbidity and mortality being reported in districts that also reported high morbidity and mortality of human diseases [12]. Despite the United States’ National Aeronautics and Space Administration (NASA) having issued an early warning for possible RVF epidemic in the horn of Africa in August 2006 approximately 3 months prior to the epidemic, no substantive interventions were carried out either by the regional governments or the international partners until confirmation of the epidemic in December 2006 [10,17]. As the outbreak unfolded with several geographically distinct sub-epidemics occurring over time, the response by the government of Kenya and international partners was hampered by the lack of information identifying areas that were susceptible to the RVF outbreak. The RVF risk maps developed here should enable the government of Kenya to have an evidence-base from which it can respond to a RVF epidemic warning as well as develop a long-term RVF prevention and control programs that target at-risk districts for optimal utilization of limited resources. Both the semi-quantitative and statistical modeling methodologies generated comparable RVF risk maps (Figs 1 and 3) that identified similar high-risk and low-risk regions of the country.

Recent RVF epidemiology indicates that certain permissive ecologies are more vulnerable to the recurrent disease epidemics than others, with the virus being endemic in these areas and being activated, amplified in livestock population and spread to infect humans during the El Nino years [18,19]. The amplified virus is spread through movement of livestock, mosquitos or humans during an epidemic, although banning livestock movement may not prevent RVF outbreaks in permissive ecosystems containing resident virus [18]. The control measures available for RVF during the threat of an epidemic include enhanced surveillance, public education, livestock vaccination, and vector control. Once an epidemic has been confirmed in a region, the same control measures except livestock vaccination are applied. Outbreak control measures including public health education need to be implemented in all areas including low-risk areas. If applied before the epidemic, targeted surveillance and livestock vaccination can be effective in either preventing an outbreak or mitigating the effects of one if it happens. It is likely that vaccinating all livestock in high risk areas that represents 30% of the national livestock population may be effective in preventing an RVF epidemic. However, it is important to emphasize that vaccinating only selected high risk areas, may not prevent outbreaks in other vulnerable districts.

Our study suggested that specific geologic and geographic factors were associated with high risk divisions, including presence of certain soil types, less than 100mm average annual rainfall during non-El Niño years, altitude below 1000 m, and densely bushed areas, high NDVI values and presence of agri-sparse vegetation. There was an ordinal reduction in risk of RVF with increasing altitude. Linkages between altitude and RVF risk have not been described. However, changes in altitude are closely associated with varying ecological conditions (demonstrated by changes in host and vector diversity, climate and physical features such as moisture content) that might be responsible for the variation in the risk levels observed. Ecosystems in low altitudes, for instance, experience irregular climate patterns that increase turnover rates of livestock and wildlife populations, compromising the maintenance of appreciable levels of herd immunity. The positive association between soil types: vertisols, solonertz and luvisols with high risk of RVF epidemics may be associated with the poor draining properties of these soils, allowing them to hold water for a longer time period compared to the other soil types. Anecdotal observations also suggest that these poor draining properties allow these soils to retain high moisture levels during the dry season to enable the survival of infected eggs of floodwater *Aedes* species that act as the primary reservoirs of RVFV. To verify these suggestions, data on mosquito profiles in various districts in Kenya would be required. These data are currently being collected in one of our research projects. Other factors that were significant in the model include NDVI and temperature. High, persistent rainfall allowed growth of abundant vegetation indicated by the high NDVI values during the high risk periods [6]. The exact causal relationship between these predictive factors and RVF disease require further investigation.

This study was subject to certain limitations. First, systematic disease surveillance that would provide accurate prevalence of RVF in each district and division during previous outbreaks or inter-epidemic periods, as well as spatial extent of the outbreaks was unavailable. The data to estimate the outcome (extent of disease in humans and animals) is therefore based on the 2006–06 epidemic only. However, RVF clinical disease in animals, particularly in an outbreak situation, is well recognized in Kenya among animal health professionals and animal herders and it is unlikely that a large number of cases were missed [20]. Whereas the retrospective surveillance data used to assess the exposure risk were collected through passive surveillance, the addition of information from expert group discussion drawn from 47 long-serving animal and public health expert from all regions of the country ensure that divisions that may have lacked official disease reports were assessed. On assessment of predictive factors of high risk districts and divisions, critical data on mosquito profiles for each district/division is not yet available.

The availability of a RVF risk map for Kenya provides an important tool for use in developing prevention and control measures against this devastating disease and for mitigation of forecasted epidemics. The map provides a roadmap for; (i) deciding where to establish sentinel surveillance among humans and livestock for early warning in compliance with the WHO international health regulations, (ii) setting up long-term disease prevention and control programs in livestock such as vaccination and public education; (iii) initiate mitigating actions in response to a forecasted epidemic; and (iv) raising the suspicion index and setting up appropriate diagnostics in human health facilities to look for cases during epidemic and inter-epidemic periods. The identification of geologic and geographic predictors of regions at high risk is only the beginning of this important research area. The risk map will also be factored into the RVF forecasting model by NASA and other existing regional RVF prediction tools to add granularity to the RVF risk predictions in Kenya. Finally, the risk map and identification of predictive factors for high risk will likely inspire other countries within the RVF ecological zones to develop risk maps for their respective countries.
